# How to Assess Health Gains

**DOI:** 10.3390/healthcare13070832

**Published:** 2025-04-05

**Authors:** Giovanni Guarducci, Gabriele Messina, Chiara Siragusa, Jolanda Gurnari, Anna Maria Gentile, Nicola Nante

**Affiliations:** 1 Post Graduate School of Public Health, University of Siena, 53100 Siena, Italy; gabriele.messina@unisi.it (G.M.); nicola.nante@unisi.it (N.N.); 2 Local Health Authority of Ferrara, 44121 Ferrara, Italy; 3 Department of Molecular and Developmental Medicine, University of Siena, 53100 Siena, Italy; chiara.siragusa1@student.unisi.it; 4 Healthcare Management, San Michele Private Hospital, 17031 Albenga, Italy; j.gurnari@student.unisi.it; 5 General Management, San Michele Private Hospital, 17031 Albenga, Italy; direzione@clinicasanmichele.it

**Keywords:** patient-centered care, health outcomes, health-related quality of life, quality-adjusted life years, cost–utility

## Abstract

**Background/Objectives**: As life expectancy rises and the epidemiological landscape of chronic diseases evolves, the necessity to assess and improve the overall health status of the population becomes increasingly fundamental. Therefore, evaluating health gains is a crucial challenge for modern health systems, particularly in the context of limited resources and increasing demand for services. The aim of this study is to assess health gains and their costs, with data provided by a private Italian clinic. **Methods**: We conducted a retrospective study on 129 patients admitted between June 2020 and August 2023 in a solvent ward for short-term planned hospitalization. The patients completed the EQ-5D-5L questionnaire at both admission and discharge. Quality-Adjusted Life Years (QALYs) were estimated based on the difference in EQ-5D-5L scores between discharge and admission, assuming that health gains persist up to two years post-discharge. Through QALYs value and hospitalization cost, a cost–utility analysis was performed. Descriptive and statistical analyses were carried out using STATA SE/14.0 software. **Results**: Of the studied sample, 55% was female, the median age was 81 [11] years old, and the median length of stay (LoS) was 16 [16] days. The patients gained, in median, 0.33 [0.38] in QALY, which was higher for males (0.35 [0.34]) than females (0.29 [0.45]). The QALY gained was greater for the non-geriatric patients (0.41 [0.42]) than geriatric ones (0.32 [0.38]) (*p* > 0.05). Those with a longer LoS showed a higher gain in QALY (0.35 [0.42]) than those with a shorter one (0.23 [0.29]) (*p* < 0.05). The cost per QALY gained was, in median, EUR 14,337, which was lower in males (EUR 13,803), in non-geriatric patients (EUR 13,743), and in patients with a shorter LoS (EUR 10,670) (*p* > 0.05). **Conclusions**: Although QALY gains differed among the groups, the median cost per QALY remained consistent. These results highlight the need for targeted interventions to optimize resource allocation, both by integrating data into allocation strategies and by employing a multidisciplinary approach to tailor interventions.

## 1. Introduction

The development of civilization has led to an increase in life expectancy, making the elderly population increasingly high over the past decades. This shift has transformed epidemiology, significantly increasing the burden of chronic diseases in terms of morbidity, disability, and mortality [1,2]. This phenomenon represents one of the most significant and complex challenges that healthcare systems must face, necessitating the development of different approaches in both patient management and care. In this context of growing demand for healthcare services, the use of tools that allow for the measurement of the quality of healthcare services provided becomes even more essential [3,4]. Quality can be assessed subjectively, based on the perceptions, expectations, and demands of various stakeholders. However, this results in a lack of consensus in defining quality across different medical fields (e.g., ophthalmology, oncology, urology, and geriatrics). The different perspectives of stakeholders can complicate the measurement and improvement of healthcare service quality [5]. There are multiple challenges in assessing the quality of healthcare services. First, there is a need for standardization of evaluation criteria to ensure meaningful comparability between different healthcare facilities and medical practices. This requires interdisciplinary dialogue and collaboration among healthcare professionals, administrators, patients, and other actors involved in the care delivery process. Another crucial aspect is the implementation of quality assessment measures based on scientific evidence. This involves the use of clinical data, patient outcomes, and indicators to evaluate the effectiveness and appropriateness of medical practices. Integrating this evidence into quality assessment can guide the adoption of best practices and improve clinical outcomes [6,7]. Additionally, it is essential to consider patients’ perspectives in assessing the quality of healthcare services. Patient experience and satisfaction are crucial elements to consider, as they reflect the impact of care—especially for long-term chronic conditions—on daily life and overall well-being. Collecting patient feedback and analyzing their needs and preferences can inform the continuous improvement of healthcare services [8,9]. At the same time, it is important to balance the focus on clinical outcomes with a cost analysis. The financial sustainability of healthcare systems depends on the ability to provide high-quality care in an efficient and economically viable manner. Therefore, the assessment of healthcare service quality should also include evaluations of effectiveness, efficiency, accessibility, and equity in care delivery [10,11]. Nowadays, the use of cost–utility analysis has received increasing attention, given the need for effective tools to guide resource allocation decisions in the healthcare sector. International studies have highlighted that evaluation based on QALYs provides a useful framework for comparing the effectiveness of interventions, particularly in mitigating the growing economic pressure due to an aging population [12,13]. However, the literature is heterogenous, as most research focuses on public facilities, while few studies have concentrated on the private sector, where cost dynamics and resource management exhibit particular characteristics [14,15]. To date, we know that at the European level average cost per QALY ranges from EUR 18,500 in public systems to EUR 16,200 in private ones, which varies significantly among Western European countries, with ranges from EUR 10,000 to EUR 45,000 [13,14]. In this context, adding a perspective on the private sector becomes essential to provide evidence that can contribute to more informed decisions, not only from a clinical perspective, but also from an economic standpoint. This allows for outlining potential reform scenarios, highlighting not only the technical challenges in measuring health gain and its cost [16], but also the implications for hospital management, which could lead to a significant improvement in the quality of care and operational efficiency [17]. Therefore, the present analysis seeks to verify whether and how the cost–utility approach in the private sector produces results that can either challenge or reinforce the models currently predominant in the healthcare system. For this reason, our study aims to evaluate both the health gain provided by hospitalization in an Italian private clinic and its associated cost.

## 2. Materials and Methods

### 2.1. Study Design, Setting, Data Collection, and Inclusion Criteria

A retrospective study with prospective purposes was conducted on 129 patients admitted to the solvent ward of a private clinic in Liguria between June 2020 and August 2023. This clinic has 100 beds, but most of its activity is dedicated to intensive post-surgical orthopedic rehabilitation in agreement with National Health Service (NHS). Health data were collected from paper medical records by clinicians and anonymized before being provided to epidemiologists for analysis. In the present study, we included all patients admitted to the solvent ward with a length of stay between 7 and 60 days and an age below 90 years. We excluded patients admitted under agreement with the NHS.

### 2.2. EQ-5D-5L

The EQ-5D-5L questionnaire is a generic measurement tool used to estimate health-related quality of life (HRQoL). The patient’s perceived health is measured through five questions exploring the following dimensions: mobility, self-care, daily activities, pain and discomfort, anxiety, and depression. Each question allows for a response across five severity levels. An algorithm sums the response scores, producing a final total score known as the EQ index. Administering the questionnaire at different time points enables the measurement of potential health gain or loss over time. This tool is, therefore, useful in evaluating healthcare outcomes and the effectiveness of medications or other treatments [18]. At both admission and discharge, the European Quality of Life 5 Dimensions 5 Level (EQ-5D-5L) questionnaire was completed autonomously by the patients included. For each patient, the difference between the score recorded at discharge and the one at admission was calculated.

### 2.3. Cumulative Illness Rating Scale (CIRS)

The CIRS is a standardized tool that provides an as objective as possible measure of a patient’s health status. The patient’s health profile is outlined using a final score based on medical history, symptomatology, and physical examination. The health status of various organs and systems is assessed by examining the severity level across fourteen categories. Each severity level corresponds to a specific score, as follows: absent—1, mild—2, moderate—3, severe—4, very severe—5. The mean score of the first 13 categories defines the Severity Index (SI), while the sum of the categories scoring 3 or higher (excluding psychiatric disorders) constitutes the Comorbidity Index (CI) [19]. In this study, to outline the “objective” health profile, the CIRS was completed at admission by the “Case Manager” physician after the collection of medical history and an objective examination. In addition, the CIRS was also calculated at discharge to assess any changes.

### 2.4. Individual Care Plan

The Problem-Oriented Individual Care Plan requires that, upon hospital admission, the following data are recorded on a dedicated card for each patient: demographic information, a summary of medical history, the reason for hospitalization, clinical parameters related to the conditions being managed, the treatment goals, the expected length of stay, the EQ-5D-5L values and its EQ index, the Severity Index and Comorbidity Index from the CIRS scale, and the Body Mass Index. This card is best described in a previously published paper [20].

### 2.5. QALY and Cost–Utility Analysis

The QALY (Quality-Adjusted Life Years) [21] was calculated using the following formula:

QALY = U × T

where:

U (utility) = change in EQ index.

T (time) = years.

The utility value (U) was obtained from the difference in EQ index between discharge and admission. The time variable (T) was 2 years, assuming an accumulation of health benefits in the 2 years following hospitalization (until it reaches the Italian life expectancy [22,23]). Additionally, each year, the utility value must be discounted by 3.5%, as recommended by NICE (National Institute for Health and Care Excellence) [23,24]. To calculate the cost of health gain per patient, the daily hospitalization cost (EUR 250.00 per day) was considered.

Based on these factors, the cost–utility analysis (CUA) was conducted by relating each patient’s hospitalization cost to the QALY gained by the patient, as follows:

CUA = Cost/QALY

where:

Cost = patient’s total cost per hospitalization.

QALY = patient’s QALY gained.

The hospitalization costs, the QALY value, and its cost were stratified by gender, age, BMI, length of stay, and clinical severity (SI of CIRS scale).

### 2.6. Statistical Analysis

STATA SE/14.0 software (StataCorp LLC, College Station, TX, USA) was used to perform various statistical tests. The Shapiro–Wilk test was applied to assess the normal distribution of the data. For descriptive analysis, the median and interquartile range (IQR) were obtained. The Mann–Whitney test was used to evaluate the differences between male and female patients, while the Wilcoxon test was employed to compare values measured at two different time points. Finally, Dunn’s test and the Mann–Whitney test were conducted to determine whether significant differences existed in both QALY and the resulting CUA across the analyzed groups. Statistical significance was set at 95% (*p* < 0.05).

## 3. Results

Table 1 provides a description of the study sample. Among the 129 patients (56.6% female), the majority were geriatric (95.3%), with a median age of 81 [11] years. The median Body Mass Index (BMI) was 25.5 [5.0], with most patients being overweight (40.3%), followed by those of normal weight (38.7%), those with Grade I obesity (12.4%), underweight (7.0%), and those with Grade II obesity (1.6%). The median length of stay was 16 [16] days, with 60.5% of patients exceeding this duration.

Table 2 shows the median values of the five dimensions of the EQ-5D-5L at admission and discharge, stratified by sex, age, BMI, and length of stay. For the “Mobility (M)” dimension, a significant improvement (*p* < 0.05) was observed between admission and discharge for the entire study sample, except for the non-geriatric patients and those with Grade II obesity. Regarding “Self-Care (S-C)”, a significant improvement (*p* < 0.05) was noted for the overall sample and for patients with a length of stay exceeding 16 days. For the “Daily Activities (D-A)” dimension, a significant improvement (*p* < 0.05) was observed across the entire sample, except for the underweight patients, those with Grade II obesity, and those with a hospital stay shorter than 16 days. For both the “Pain and Discomfort (P-D)” and “Anxiety and Depression (A-D)” dimensions, a significant improvement (*p* < 0.05) between admission and discharge was found for the entire study sample, except for the non-geriatric patients and those with Grade II obesity.

Table 3 shows the EQ index values at admission, discharge, and their difference, stratified by sex, age, BMI, and length of stay. It was observed that females had higher EQ index values than males at both admission and discharge. At both time points, patients under 65 years old had a higher EQ index (0.531 at admission and 0.740 at discharge) compared to those over 65 years old (0.381 at admission and 0.567 at discharge). The EQ index delta was also higher for younger patients (0.142) compared to older ones (0.120). According to BMI categories, at admission, the highest EQ index was recorded in patients with Grade II obesity, followed by overweight, normal-weight, underweight, and Grade I obese patients. However, at discharge, the highest EQ index was found in Grade II obese patients, followed by Grade I obese, overweight, normal-weight, and underweight patients. The patients with a shorter length of stay (<16 days) had higher EQ index values at both admission and discharge compared to those with a longer hospitalization time. The difference between admission and discharge was significant for all analyzed categories, except for non-geriatric patients, underweight patients, and those with Grade II obesity (*p* < 0.05). Above-median EQ index values were observed in males, younger patients, overweight individuals, Grade I and II obese patients, and those with a length of stay longer than 16 days.

Table 4 presents the median values of the CIRS scale, along with the corresponding Severity Index and Comorbidity Index, stratified by sex and length of stay, both at admission (Adm.) and discharge (Dis.). According to sex differences, females showed significantly higher values than males in the “cardiac” category, both at admission and discharge. Conversely, they had significantly lower values in the categories of “eyes, ears, nose, pharynx, and larynx”, “upper gastrointestinal tract”, “genitourinary system”, “endocrine-metabolic system and breast”, and “psychiatric and behavioral disorders” (*p* < 0.05). Regarding the length of stay, patients with a shorter hospitalization time had significantly lower values in the “vascular-hematopoietic” and “psychiatric and behavioral disorder” categories (*p* < 0.05). Finally, patients with a longer hospitalization time had significantly higher Comorbidity Index values (*p* < 0.05).

Table 5 presents the main issues addressed in the Individual Care Plans (PAI) for the 129 patients included in this study. The most frequently reported issue was pain (measured in Numeric Pain Rating Scale (NRS), followed by difficulties in performing daily activities (ADLs), mobility impairments, anemia, and wound management. Significant improvements were observed in pain management, daily activities, mobility, anemia, and wound condition (*p* < 0.05).

Table 6 presents the health gain expressed in QALY, hospitalization reimbursement, and cost per QALY, stratified by sex, age, BMI, length of stay, and clinical severity. The median QALY gained by the studied patients was 0.33 [0.38], with a higher gain observed in males (0.35 [0.34]) compared to females (0.29 [0.45]). The patients under 65 years old experienced a greater QALY gain (0.41 [0.42]) than the geriatric patients (0.32 [0.38]). Regarding BMI categories, the highest QALY gains were observed in Grade II obese patients (0.44 [0.34]), followed by Grade I obese (0.40 [0.43]), overweight (0.31 [0.41]), normal-weight (0.30 [0.48]), and underweight patients (0.18 [0.32]). The patients with a longer-than-median hospital stay had a greater QALY gain (0.35 [0.42]) compared to those with a shorter hospitalization stay (0.23 [0.29]) (*p* < 0.05). Patients with a Severity Index above the median showed a higher QALY gain (0.29 [0.19]) compared to those with lower clinical severity (0.29 [0.40]). The median hospitalization reimbursement was EUR 4074, with a higher reimbursement for females (EUR 4190) compared to males (EUR 3957). Regarding age, there was a slight difference in reimbursement between patients over 65 years old (EUR 3957) and those under 65 years old (EUR 3841). Analyzing BMI categories, the highest hospitalization reimbursement was observed in the underweight patients (EUR 4888), followed by Grade II obese (EUR 4423), Grade I obese (EUR 4191), normal-weight (EUR 3958), and overweight patients (EUR 3725). The patients with a longer hospital stay had a higher hospitalization reimbursement (EUR 6751) compared to those with a stay of less than 16 days (EUR 3026). For the patients with a higher Severity Index, hospitalization reimbursement was slightly higher (EUR 4190) compared to those with a lower Severity Index (EUR 3724). The calculated cost per QALY was EUR 14,337, with higher costs for females (EUR 14,945) compared to males (EUR 13,804). Regarding age, there was a slight difference in cost per QALY between patients over 65 years old (EUR 14,338) and those under 65 years old (EUR 13,744). Stratifying patients by BMI, the cost per QALY was EUR 14,474 for underweight patients, EUR 14,456 for Grade II obese patients, EUR 14,344 for overweight patients, EUR 14,228 for normal-weight patients, and EUR 7948 for Grade I obese patients. The patients with a lower Severity Index had a slightly higher cost per QALY (EUR 14,187) compared to those with a higher Severity Index (EUR 14,344).

## 4. Discussion

The increase in life expectancy has led to the need to measure health-related quality of life (HRQoL), an aspect that must be considered when evaluating healthcare outcomes. The aim of this study was to measure the health gain produced by a private clinic. For this purpose, the EQ-5D-5L questionnaire, widely used in the measurement of HRQoL in health care [25], was used, which was subsequently converted into QALY. In the study sample, 95.34% of the individuals were over 65 years old, making it homogeneous in terms of age. However, it was heterogeneous in terms of BMI, including underweight, normal-weight, overweight, and obese patients, without a clear predominance of any category. Stratifying the sample by BMI, as carried out in other studies [26,27,28], did not affect the results obtained from age stratification. The most impaired dimension in EQ-5D-5L remained mobility and daily activities, and the same was observed for sex stratification. These findings suggest that neither sex nor BMI are the primary factors influencing these aspects of quality of life, but rather age. These results align with those of other studies on geriatric patients [27,29,30], which have demonstrated the negative impact of aging on mobility and daily activities, with a lesser effect on anxiety and depression. Other studies considering all age groups from 18 years onward [16,31,32,33] report different results, indicating that anxiety and depression are the most prevalent issues. Patients with longer hospital stays (≥16 days) showed improvements in all EQ-5D-5L dimensions at the end of treatment, except for self-care and anxiety/depression. However, these patients were not hospitalized for psychiatric conditions, so treatments focused on other priorities. Beyond the results of the individual dimensions of the EQ-5D-5L questionnaire, the most important outcome was the EQ index of each patient and its variation between admission and discharge. Regarding age, as also found in other studies [32,33,34,35], younger patients had higher EQ index values at both admission and discharge compared to geriatric patients. However, no major differences were observed in the EQ index delta values between the two age groups. All patients, therefore, achieved a similar health gain in terms of HRQoL, regardless of age. When stratifying patients by sex, it was found that males in this sample achieved a greater health gain despite starting with lower EQ index values than females. This differs from what is observed in the literature in studies investigating perceived health in the general population across all age groups [30,34,35]. When stratifying patients by BMI, differences in EQ index delta were observed, with first-degree obese patients achieving the highest gain, followed by overweight patients, and finally normal-weight individuals. When examining the two groups based on length of stay, patients with longer hospital stays (≥16 days) achieved a greater health gain, despite starting with lower EQ index values. To objectively define health profiles, the Cumulative Illness Rating Scale (CIRS) was used, which has already been shown to be suitable in hospital settings, as demonstrated in other studies [36,37,38,39]. Data collection revealed that males in the sample were characterized by greater management complexity, as they had higher values of both the Severity Index and the Comorbidity Index. Higher severity levels were found in the otorhinolaryngological district, the upper gastrointestinal system, the endocrine-metabolic system, and the psychiatric and behavioral domains. In females, greater complexity was observed only in the cardiac and genitourinary systems at admission. However, at discharge, an improvement in the genitourinary system was observed, bringing their CIRS scores in line with those of males, thanks to the effectiveness of the clinic’s treatments. Improvements in the vascular-hematopoietic system were observed in both sexes in patients with a hospital stay of 16 days or more. To optimize patient management, the Problem-Oriented Individual Care Plan (PO-ICP) was adopted, which involves listing the main health problems of each patient to define and agree on the diagnostic-therapeutic objectives to be pursued. In this sample, the most frequently observed issue was pain, followed by difficulties in daily activities, mobility impairment, anemia, and wound management. The collection of these data was useful both for clinical management and care, as well as for ensuring greater transparency in data collection [40]. The PO-ICP was also useful for measuring the effectiveness of the healthcare services provided, as statistically significant improvements were observed in all major identified problems, as already shown [20]. The concept of achieving clinical outcomes includes both an increase in life expectancy and an improvement in quality of life. These two concepts of quantity and quality are combined in QALY, which was, therefore, calculated for each patient and represents the measure of health gain. This study found that males achieved greater health benefits, gaining four additional months in excellent health, one more than females. Younger patients showed a greater QALY gain, with five months in full health compared to three and a half months for geriatric patients. Considering BMI categories, obese patients appeared to gain the most, with about five months in full health, one month more than overweight patients and three months more than underweight patients. Certainly, further research will certainly need to clarify a possible role of BMI in health gain, as any confounding factors, such as age, could have an impact on it, or the other BMI classes may have more complex health conditions. Patients with longer hospital stays had higher QALY values, showing a greater health gain than those with shorter hospital stays, with a difference of three months. The most critical patients, with a higher Severity Index, gained four months, while those with a lower Severity Index gained three and a half months. The median QALY gain was 0.32 (four months), which is different to the values found in other studies [35,41]. However, it is important to note that although some differences between groups are not significant, they exceed the threshold of the minimum clinically important difference (equal to 0.03 QALY [42,43]), suggesting a potential clinically relevant impact, despite the lack of statistical significance. Additionally, an economic evaluation was also conducted because cost–utility analyses, which compare costs with QALYs, are important for objectively determining the effectiveness of care. For this reason, the cost per unit of QALY was calculated. The results show that the calculated cost to obtain one QALY, meaning one year in full health, was EUR 14,337, well below the maximum QALY threshold of EUR 40,000 established by the guidelines proposed by the Italian Association of Health Economics (AIES) [44,45]. The results obtained highlight a better performance of the studied clinic compared with that of previous years [20].

### Limitations

Although this methodological work provides important insights into the assessment of hospital care outcomes (health gain), it has some limitations. This work focuses on a single care setting and a small specific sample (mostly geriatric patients), which may limit the generalizability of the results. In addition, in the absence of a control group, the observed results may not be attributable solely to hospital care.

## 5. Conclusions

The measurement tools employed allowed the conversion of both patient-reported and clinical assessments (through EQ-5D-5L and CIRS, respectively) into comparable numerical values. These measurements, together with the problem-oriented approach implemented via Individual Care Plans, facilitate patient management optimization and outcome evaluation. Notably, the CIRS scale appeared less sensitive in gauging the effectiveness of short-term hospital stays and may be better suited for long-term evaluations. Although it is recognized as a valuable risk adjustment tool, its limited variation in our homogeneous elderly sample may have diminished its impact in this analysis. The EQ-5D-5L questionnaire provided the essential data to determine health gains in terms of QALYs, and, in our sample, only the length of hospitalization showed a significant association with the measured health gains. Furthermore, male patients appeared to report greater health benefits, and the few patients under 65 years of age demonstrated better outcomes. The cost–utility analysis revealed a cost per QALY gained that remains below the limits set by the AIES, supporting the appropriateness and potential effectiveness of the services provided by the healthcare facility. Moreover, when compared to a previous study conducted on cases from 2018 to 2020, the cost per QALY gained in our sample was halved. This improvement is likely attributable to enhanced information management and, perhaps, more effective patient management practices. In conclusion, the observed data indicate that hospital admissions are quantifiable and may be further stratified by each treated condition. The economic valuation of these ‘health gains’ offers a useful framework for resource allocation and healthcare performance improvement. However, while a significant association between certain variables (e.g., length of hospitalization) and health gains was observed, confounding factors cannot be entirely excluded. Therefore, policy makers are encouraged to consider these data as part of a wider evidence base when designing allocation strategies, particularly focusing on populations that appear to benefit most, while further studies are recommended to reinforce these findings. For clinicians, integrating outcomes within a multidisciplinary context could support a more tailored therapeutic approach.

## Figures and Tables

**Table 1:** Studied sample of a cohort of 129 patients.

Patients	N°	%
**TOTAL**	129	100%
**Sex**		
Male	56	43.4%
Female	73	56.6%
**Age** (median 81 [11])		
<65 years old	6	4.7%
≥65 years old	123	95.3%
***BMI*** (median 25.5 [5.0])		
Underweight	9	7.0%
Normal weight	50	38.7%
Overweight	52	40.3%
Obese I Class	16	12.4%
Obese II Class	2	1.6%
**Length of stay** (median 16 [16])		
<16 days	51	39.5%
≥16 days	78	60.5%

**Table 2:** Median values of the five dimensions of EQ-5D-5L (admission and discharge), stratified by sex, age, BMI, and length of stay.

	Admission					Discharge				
	M	S-C	D-A	P-D	A-D	M	S-C	D-A	P-D	A-D
**Sex**										
Male	4 [1] *	3 [1]	4 [1] *	3 [1] *	2 [1]	3 [1] *	3 [1]	3 [1] *	2 [1] *	2 [1]
Female	4 [1] *	3 [1]	3 [1] *	3 [2] *	2 [2]	2 [1] *	3 [1]	3 [1] *	2 [1] *	2 [1]
**Age**										
<65 years old	3 [2]	3 [1]	4 [1] *	3 [1]	2 [1]	2 [1]	2 [1]	2 [1] *	2 [1]	2 [1]
≥65 years old	4 [1] *	3 [1]	4 [2] *	3 [1] *	2 [2] *	3 [1] *	3 [1]	3 [1] *	2 [1] *	2 [1] *
***BMI***										
Underweight	3 [2] *	3 [1]	3 [1]	3 [1] *	3 [1] *	2 [1] *	3 [1]	3 [1]	2 [1] *	2 [1] *
Normal weight	4 [1] *	3 [1]	4 [1] *	3 [1] *	2 [1] *	3 [1] *	3 [1]	3 [1.5] *	2 [1] *	2 [1.5] *
Overweight	4 [1] *	3 [1]	4 [1] *	3 [1] *	2 [1] *	3 [1] *	3 [1]	3 [1] *	2 [1] *	2 [1] *
Obese I Class	3 [1] *	3 [1]	4 [1] *	3 [1] *	2 [2] *	2 [1] *	2 [1]	3 [0.5] *	2 [1] *	2 [1.5] *
Obese II Class	3 [1]	3 [1]	3 [1]	2 [1]	1 [1]	2 [1] *	2 [1]	2 [1]	1 [0]	1 [0]
**Length of stay**										
<16 days	3 [1] *	3 [1]	3 [1]	3 [1] *	2 [1]	2 [1]	3 [1]	3 [1]	2 [1] *	2 [1]
≥16 days	4 [1] *	4 [1] *	4 [1] *	3 [1] *	2 [1] *	3 [1] *	3 [1] *	3 [1] *	2 [1] *	2 [2] *
**Total**	4 [2] *	3 [2] *	4 [2] *	3 [1] *	2 [1] *	3 [1] *	3 [1] *	3 [1] *	2 [1] *	2 [1] *

* *p* < 0.05 between admission and discharge.

**Table 3:** Median EQ index values (admission, discharge, and delta), stratified by sex, age, BMI, and length of stay.

	Admission	Discharge	Delta
**Sex**			
Male	0.378 [0.276]	0.56 [0.167]	0.158 [193] *
Female	0.423 [0.377]	0.592 [0.247]	0.098 [0.235] *
**Age**			
<65 years old	0.531 [0.225]	0.740 [0.214]	0.142 [0.302]
≥65 years old	0.381 [0.335]	0.567 [0.197]	0.120 [0.204] *
***BMI***			
Underweight	0.378 [0.551]	0.558 [0.439]	0.039 [0.173]
Normal weight	0.387 [0.318]	0.565 [0.232]	0.099 [0.233] *
Overweight	0.445 [0.303]	0.570 [0.119]	0.126 [0.199] *
Obese I Class	0.359 [0.676]	0.586 [0.108]	0.173 [0.273] *
Obese II Class	0.578 [0.314]	0.812 [0.130]	0.234 [0.184]
**Length of stay**			
<16 days	0.487 [0.442]	0.586 [0.222]	0.075 [0.229] *
≥16 days	0.310 [0.277]	0.555 [0.129]	0.190 [0.190] *
**Total**	0.406 [0.322]	0.567 [0.197]	0.120 [0.224] *

* *p* < 0.05 between admission and discharge.

**Table 4:** Median values (admission and discharge) of the CIRS scale stratified by sex and length of stay.

CIRS Categories	Male		Female		Length of Stay < 16		Length of Stay ≥ 16	
	Adm.	Dis.	Adm.	Dis.	Adm.	Dis.	Adm.	Dis.
**Heart**	1 [2] *	1 [2] *	3 [2] *	3 [2] *	1 [2]	1 [2]	2 [2]	2 [2]
**Blood pressure**	3 [2]	3 [2]	3 [2]	3 [2]	3 [1]	3 [1]	3 [2]	3 [2]
**Vascular**	2 [1]	2 [1]	2 [2]	2 [2]	2 [1] *	2 [1] *	2 [2] *	1 [2] *
**Respiratory**	1 [0]	1 [0]	1 [1]	1 [1]	1 [1]	1 [1]	1 [1]	1 [1]
**Sense organs**	2 [1.5] *	2 [1.5] *	1 [1] *	1 [1] *	2 [1]	2 [1]	1 [1]	1 [1]
**Upper G.I.**	3 [0] *	3 [0] *	1 [2] *	1 [2] *	3 [2]	3 [2]	3 [2]	3 [2]
**Lower G.I.**	1 [0]	1 [0]	1 [0]	1 [0]	1 [0]	1 [0]	1 [0]	1 [0]
**H-P**	1 [0]	1 [0]	1 [0]	1 [0]	1 [0]	1 [0]	1 [0]	1 [0]
**Renal**	1 [0]	1 [0]	1 [0]	1 [0]	1 [0]	1 [0]	1 [0]	1 [0]
**Genitourinary**	1 [1] *	1 [1] *	2 [2] *	1 [2] *	2 [1]	1 [1]	1 [1]	1 [1]
**MS and skin**	4 [0]	3 [0]	4 [1]	3 [1]	4 [1]	3 [1]	4 [1]	3 [1]
**Neurological**	1 [0]	1 [0]	1 [0]	1 [0]	1 [0]	1 [0]	1 [0]	1 [0]
**Endocrine**	3 [2] *	3 [2] *	1 [2] *	1 [2] *	2 [2]	2 [2]	3 [2]	3 [2]
**Psychiatric**	4 [3] *	4 [3] *	1 [3] *	1 [3] *	1 [3] *	1 [3] *	3 [3] *	3 [3] *
**Comorbidity Index**	4.50 [2]	4.50 [2]	4 [2]	4 [2]	4 [1] *	4 [1] *	5 [2] *	5 [2] *
**Severity Index**	1.92 [0.38]	1.92 [0.38]	1.85 [0.35]	1.85 [0.35]	1.85 [0.31]	1.85 [0.31]	1.92 [0.46]	1.92 [0.46]

* *p* < 0.05 between the two groups. G.I. = gastro-intestinal; H-P = hepatic-pancreatic; MS = musculoskeletal.

**Table 5:** Principal problems of 129 patients taken care of in the Individual Care Plan.

Problem	N° Observation	Measuring Tool	Admission Value	Discharge Value	Significant Improvement
**Pain**	108	Scala NRS	6 [2]	3 [1]	*p* < 0.05
**ADLs**	101	Scala Barthel	65 [40]	85 [30]	*p* < 0.05
**Walking difficulties**	101	Scala Tinetti	18 [13]	23 [8]	*p* < 0.05
**Anemia**	27	Haemoglobin (g/dL)	10.7 [1.4]	11.3 [1.6]	*p* < 0.05
**Surgical wound**	17	C-reactive protein and Photo	3.4 [2.0]	1.1 [2.3]	*p* < 0.05

**Table 6:** QALY values, hospitalization remuneration, and the cost per QALY stratified by sex, age, BMI, days of hospitalization, and clinical severity.

Patient	Median Gain in QALY	Hospitalization Costs (EUR)	*QALY*	
			Median Cost (EUR)	IQR (EUR)
**Total**	0.32127 [0.37807]	4.074	14.337	20.945
**Sex**				
Male	0.34642 [0.34641]	3.957	13.804	17.658
Female	0.2924 [0.44885]	4.190	14.945	33.918
**Age**				
<65 years old	0.41346 [0.41532]	3.841	13.744	15.164
≥65 years old	0.32127 [0.38366]	3.957	14.338	21.025
***BMI***				
Underweight	0.18252 [0.32034]	4.888	14.474	88.942
Normal weight	0.30358 [0.47864]	3.958	14.228	25.807
Overweight	0.31382 [0.40880]	3.725	14.344	17.147
Obese I Class	0.3995 [0.43471]	4.191	7.948	17.360
Obese II Class	0.43581 [0.34269]	4.423	14.456	2.538
**Length of stay**				
<16 days	0.22908 [0.29240]	3.026	10.671	19.407
≥16 days	0.35387 [0.41718]	6.751	15.064	18.348
**Severity Index**				
<1.92	0.28868 [0.4061]	3.724	14.344	19.660
≥1.92	0.3520 [0.40228]	4.190	14.187	19.431

## Data Availability

The data that support the findings of this study are available on request from the corresponding author. The data are not publicly available due to privacy or ethical restrictions.
